# The Psychology of Fear: the Effects of Panic Fear in Wartime

**Published:** 1917-03-24

**Authors:** Robert Armstrong-Jones

**Affiliations:** Late Medical Superintendent, Claybury County Asylum.


### THE PSYCHOLOGY OF FEAR.

### THE EFFECTS OF PANIC FEAR IN WARTIME.

**Abstract of a Paper read before the Medical Society of London.**

By HON. MAJOR SIR ROBERT ARMSTRONG-JONES, M.D. (late Medical Superintendent, Claybury County Asylum).

It is an acknowledged fact that in the whole annals of mankind the most eventful period of a nation's psychology is that during which its people is passing through the crisis of war; and the history of nations, from the earliest dawn of society, presents continuous records of warlike operations. Looked upon psychologically, war is the manifestation of a biological law, and it is the embodiment in men of a primordial and deep-rooted instinct. War is an instinct which is associated with the emotions of anger and hate, as well as of fear. Psychologists and physiologists now all teach that the emotions have very definite physical correlatives: Pawlow, Elliot, Cannon, Sherrington, Shand, and Crile have all urged this. The presentation to consciousness of visceral sensation was an early recognition. The Psalmist referred to it: "My bowels yearned within me"; "My tongue cleaved to the roof of my mouth." And we have frequent references to the organic sensations in the visceral perturbations, præcordial anxieties, rapid pulse, and "cold sweats" of shell-shock cases.

### The Desire for Flight.

A young officer, who had obtained the Military Cross for bravery, told the author that on one occasion, when alone and in danger, he was overcome by a sudden fear: "something within me seemed to pass right away." Then his body began to tremble, but, by an effort of will, this passed off. Otherwise, his feeling was to get right away from where he was. In all dangers the effect is the same, and Crile states the whole effort of the organism is to obtain an "atmosphere" of "a-nosi-association," *i.e.* protective visceral reflexes occur, and these are precisely similar to those for combat or flight. Although the action of the central nervous system is of the syndromic variety, the cortex being organised and activated to a high degree of perfection upon a united basis, yet the mind may become dissociated and any one element may predominate.

### Sensory-motor Disturbances.

Fear, especially of the vague, marginal, subconscious type, may effect this, and more so when the bodily defences are lowered by stress and insomnia. Then any sudden, overwhelming cause, especially loud noises or sudden unexpected sounds, may bring about a dissociation, as is seen in so many shell-shock cases. Witness, for instance, the deaf and dumb who are even unable to whisper, those who may be said to be "struck dumb" by fear, or those who lose their sight and are "struck blind," yet who recover suddenly. And there are those who exhibit sensory-motor disturbances, and are seen in large numbers in the various military hospitals. In regard to sensory disturbances it is the most highly evolutionised and the least organised that soonest suffer dissociative dissolutions: hearing and sight, rather than taste and smell. As to sensory-motor disturbances, we realise that consciousness is a continuous dynamic process. The normal cortex is in a state of constant nervous tension, ready, at any instant, to discharge and respond to any stimulus, the most facile track being the most ancient as well as the most elementary—*i.e.* the lower motor neurons; so that when any disturbing stimulus arises, the co-ordination of all the usual intercurrent stimuli in the cortex is affected, and a discharge occurs along the easiest routes. Not infrequently this discharge excites other areas in more distantly related centres, the form of the discharge depending on the individual temperament or upon the readiness of innately organised areas to respond.

### Stimulation by Noise.

Loud noises often act as disturbing stimuli because hearing depends upon very specific receptors, the delicate hair-cells to which the tectorial membrane resounds, and there is an intimate association between tactile and auditory sensations, as well as between motor and auditory; and any sudden stimulation of the specific auditory epithelium is capable of reacting, in a very general way, upon the mental functions. The stream of sudden stimuli to the auditory nerve must also affect its vestibular branch, which is closely connected with the static sense and with the control of movement. And although the vestibular nerves give rise to no sensations, they are nevertheless closely related to the roots of the motor-oculi nerves, as well as to the other motor centres in the medulla and cerebellum. They thus serve to keep up the bodily balance and to keep the eyes fixed upon the same point, in spite of movements of the head and body.

### The Correlation of Auditory Impulses with Movements.

All auditory impulses, as well as visual, must therefore be continuously correlated with movements, yet there is no knowledge in consciousness that these movements are made or produced during normal hearing and seeing. A large number of shell-shock cases exercise uncontrolled movements, especially when suddenly spoken to; and this fear is often imitative, and may be excited through suggestion. A soldier suffering from shell-shock was out on parole when he saw a horse fall, and suddenly he went down too, and had to be carried back. An aeroplane pilot had a false landing and fell on his head. He was blind for three hours, and at intervals completely loses sight and feels dizzy, being unable to stand or walk, although there is no organic lesion. The extreme suddenness of the symptoms is only equalled by the extreme suddenness of their disappearance, which seems to suggest the assumption of some entity "mind." And the mental experiences of the war favour a view of interactionism, rather than of parallelism, as to the relationship between mind and body. A shell-shock case, deaf and dumb, walked up Parliament Street when he was suddenly greeted by his brother, whom he had not seen for several years, and he spoke from that moment. A group of cases went to witness a transpontine melodrama, part of the play being the explosion of a mimic shell on the stage. Five of their number had to be carried back, and one became aphasic and aphonic.

### Views as to Cause of Shell-shock.

As to the cause of shell-shock, although an indescribable, unknown fear is a preliminary condition, it is impossible to dogmatise when the symptoms are so variable. Crile has shown that in every case where psychic stimuli have been applied to excess experimentally by frightening caged animals with dogs, and by worrying rabbits, and keeping animals from sleeping for long periods, definite neuronic changes, commencing with hyperchromatism and passing into chromato-lysis, have been observed in the cerebrum, cerebellum, medulla, and cord: also in the cells of the liver, thyroid, and suprarenals, so that we may be said to fear in our visceral organs as well as in our brains. At least five views have been expressed as to the cause of shell-shock: (*a*) hypothyroidism, (*b*) hyperthyroidism, (*c*) the inhalation of poisonous gases such as carbon monoxide or phosgene, which cause a disintegration of the red blood cells and consequent hæmorrhage, (*d*) a definite molecular physical injury, due to the exceedingly high pressure—sometimes positive, sometimes negative—from the explosion of shells. It is considered that the cerebro-spinal nervous system hangs loosely in a fluid bag, and any concussion would be communicated to the anterior horn cells and the fine cells of the intermediolateral tract sooner than to the posterior spinal root ganglia, which are protected by a dura mater sheath in the intervertebral spaces; and that there is consequent loss of movement rather than of sensation. This accords with experience. Also, the dissociation between the cerebro-spinal and the autonomic systems would bring about those sympathetic symptoms of dilated pupil, rapid heart, dyspepsia, precordial pains, and visceral perturbations so often seen. (*e*) There is also the sudden effect of an unexpected fear, terror, or fright acting without warning as a strong emotional shock. And there is no doubt, from experimental records, that a physiological, if not an organic, change occurs in the cytoplasm of the neurons, and such a shock would also cause this dissociation between the cerebro-spinal and autonomic systems with the symptoms already named.

### Fear Necessary to Self-preservation.

Fear connotes a mental state in which the future appears to dominate the present, whilst the actual present is a revived experience of the past, this experience being a painful one, and it is this revival that constitutes the emotion of fear. The power to experience fear is necessary to self-preservation, and it is met with in early conscious life as well as in animals; the most easily frightened member of the herd, *cæteris paribus*, having the best chance of survival. Fear acts as a tonic to some natures, and to most of our soldiers at the Front their life without danger or peril or fear would be insipid and flat. Fear is of two kinds, the sudden, "unconscious," indescribable reaction to danger, which is highly infectious; and the reasoned fear of the courageous man. It is the former that causes masses of people to experience panic, and it is this fear that is the most characteristic of shell-shock cases, who should not be treated together in groups of like cases, but, as has been wisely suggested, interspersed with "super-optimists."

### The God Pan.

It is not generally appreciated that the word "panic" is derived from the god Pan, who was awful to behold, whose voice instilled terror, and who made armies to flee. There are records of panic fears from time immemorial during wars, pestilence, revolutions, financial crises, earthquakes, and fires; but the relevant instances are those of war, as in the records of Grote, Mommsen, and Biblical history. The subject has also interested sculptors. Artists have also endeavoured to describe the emotions in attitudes characteristic of the instincts of terror, fright, horror, awe, reverence, and sympathy, and the author gave a number of instances in classical art.

Among the civil population, during any sudden crisis, the emotions of many, even of superior minds, crystallise round the feelings of the moment. More especially is this the case when danger is anticipated, as occurred among those whose minds had a psychopathic tendency during the Zeppelin raids, which only served as the occasion upon which the morbid element and the insane tendency reacted to fear.

Practically all cases of shell-shock, which the author maintains are caused through sudden, unconscious fears and awe, are quite restored if treated early.

